# 5-Bromo-4-(3,5-di­bromo-2-hy­droxy­phen­yl)-2-(piperidin-1-yl)-1,3-di­thiol-2-ylium bromide

**DOI:** 10.1107/S1600536813016048

**Published:** 2013-06-15

**Authors:** Paul Chirita, Cristian G. Hrib, Lucian M. Birsa

**Affiliations:** a Department of Chemistry, University of Craiova, 107I Calea Bucuresti, Craiova, Romania; b Chemisches Institut der Otto-von-Guericke-Universität, Universitätsplatz 2, D-39116 Magdeburg, Germany; c Department of Chemistry, "Al. I. Cuza" University Iasi, 11 Carol I Bvd, Iasi 700506, Romania

## Abstract

In the title salt, C_14_H_13_Br_3_NOS_2_^+^·Br^−^, synthesized by bromination of mesoionic 2-[2-(piperidin-1-yl)-1,3-di­thiol-2-ylium-4-yl]phenolate in glacial acetic acid, the dihedral angle between the 1,3-di­thiol­ium ring and the phenolic substituent ring is 45.9 (3)° due to the steric influence of the *ortho*-Br group on the 1,3-di­thiol­ium ring. The piperidine ring adopts a chair conformation. In the crystal, the cation and anion are linked by an O—H⋯Br hydrogen bond.

## Related literature

For applications of 1,3-di­thiol­ium salts, see: Narita & Pittman (1976 ▶); Bryce (2000 ▶); Birsa & Ganju (2003 ▶); For the structure of 2-ethyl­thio-4,5-bis­(tri­fluoro­meth­yl)-1,3-di­thiol-2-ylium hexa­chloro­stibiate, see: Frasch *et al.* (1993 ▶)
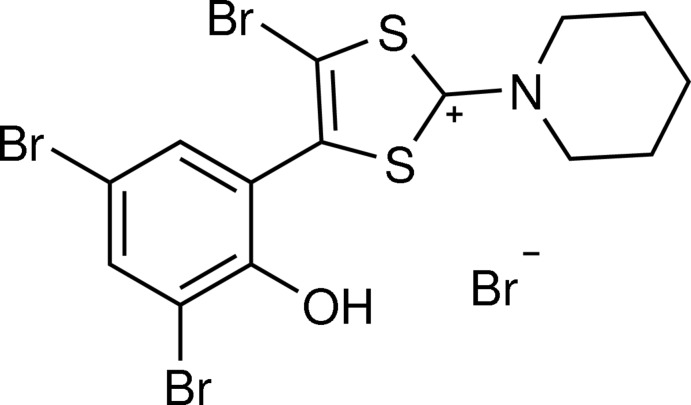


## Experimental

### 

#### Crystal data

C_14_H_13_Br_3_NOS_2_^+^·Br^−^
*M* *_r_* = 595.01
Monoclinic, 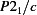

*a* = 10.484 (2) Å
*b* = 7.9240 (16) Å
*c* = 21.396 (4) Å
β = 95.16 (3)°
*V* = 1770.4 (6) Å^3^
*Z* = 4
Mo *K*α radiation
μ = 9.33 mm^−1^
*T* = 153 K
0.26 × 0.13 × 0.05 mm

#### Data collection

Stoe IPDS 2T area-detector diffractometer
Absorption correction: for a sphere [modified Dwiggins (1975 ▶)] *T*_min_ = 0.047, *T*_max_ = 0.073
19422 measured reflections
4399 independent reflections
3413 reflections with *I* > 2σ(*I*)
*R*_int_ = 0.132

#### Refinement

*R*[*F* ^2^ > 2σ(*F* ^2^)] = 0.061
*wR*(*F* ^2^) = 0.137
*S* = 1.08
4399 reflections
200 parameters
H-atom parameters constrained
Δρ_max_ = 1.52 e Å^−3^
Δρ_min_ = −1.03 e Å^−3^

### 

Data collection: *X-AREA* (Stoe & Cie, 2002 ▶); cell refinement: *X-AREA*; data reduction: *X-AREA*; program(s) used to solve structure: *SHELXS97* (Sheldrick, 2008 ▶); program(s) used to refine structure: *SHELXL97* (Sheldrick, 2008 ▶); molecular graphics: *XP* in *SHELXTL* (Sheldrick, 2008 ▶); software used to prepare material for publication: *SHELXL97*.

## Supplementary Material

Crystal structure: contains datablock(s) I, global. DOI: 10.1107/S1600536813016048/zs2264sup1.cif

Structure factors: contains datablock(s) I. DOI: 10.1107/S1600536813016048/zs2264Isup2.hkl

Supplementary material file. DOI: 10.1107/S1600536813016048/zs2264Isup3.cdx

Supplementary material file. DOI: 10.1107/S1600536813016048/zs2264Isup4.cml

Additional supplementary materials:  crystallographic information; 3D view; checkCIF report

**Fig. 1.**
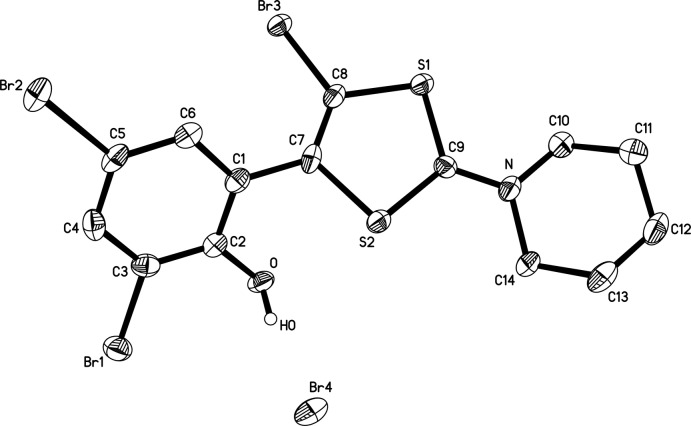
The atom numberimg scheme for the cation and anion species of the title salt. Thermal ellipsoids are drawn at the 50% probability level.

Enhanced figure: interactive version of Fig. 1

## Figures and Tables

**Table 1:** Hydrogen-bond geometry (Å, °)

*D*—H⋯*A*	*D*—H	H⋯*A*	*D*⋯*A*	*D*—H⋯*A*
O—H0⋯Br4	0.84	2.30	3.120 (5)	167

## supplementary crystallographic information

### Comment

1,3-Dithiolium salts are well known precursors of tetrathiafulvalenes (Narita & Pittman, 1976), which in turn are notable π-electron donors in organic superconductors (Bryce, 2000). Of special interest are systems where the donor moiety is linked through a σ- or π-bonded bridge to the acceptor moiety. In this context, it has been shown that 1,3-dithiolium ions can also serve as acceptor moieties in intramolecular charge-transfer systems (Birsa & Ganju, 2003). The title compound, C_14_H_13_Br_3_NOS_2_^+^ Br^-^, has been synthesized in good yield (84%), by bromination of mesoionic 2-[2-(piperidin-1-yl)-1,3-dithiol-2-ylium-4-yl]phenolate in glacial acetic acid. In this salt (Fig. 1), the benzene and 1,3-dithiolium planes form a dihedral angle of 45.9 (3) °, this deviation from planarity most likely being due to the bulky bromine substituent in the 5-position of 1,3-dithiolium ring. Moreover, no hydrogen bond was found between the phenolic O—H group and the S2 atom. Instead, a hydrogen bond between the O—H group and the bromide counter-anion is present (Table 1). Also present in the crystal is a weak intermolecular C13—H···Br2^i^ hydrogen bond [3.836 (7) Å], a short intermolecular Br3···Br4^ii^ interaction [3.3062 (11) Å] and a weak dithiolium to phenyl ring π–π interaction [minimum ring centroid separation, 3.801 (4) Å] [for symmetry code (i) -*x* + 1, *y* + 1/2, -*z* + 1/2; (ii) -*x* + 1, *y* - 1/2, -*z* + 1/2].

### Experimental

To a solution of 0.277 g (1 mmol) of 2-[2-(piperidin-1-yl)-1,3-dithiol-2-ylium-4-yl]phenolate (Birsa & Ganju, 2003) in 20 ml of glacial acetic acid, a solution of 0.15 ml (3 mmol) of bromine in 2 ml of glacial acetic acid was added dropwise. After complete consumption of the bromine the reaction mixture was poured into water and the precipitate filtered off. Crystallization from ethanol give 0.5 g (84%) of pure product as colorless crystals [m.p. 501–502 K (dec.)]. IR (ATR): ν_max_ 2946, 2764, 2551, 1567, 1523, 1439, 1248, 1229, 868, 852, 687 cm^-1^. *1*H NMR (300 MHz, DMSO-d6): *δ* = 1.77 (m, 6H, 3CH2), 3.89 (m, 4H, 2CH2-N), 7.55 (d, 4 J=2.1 Hz, 1H), 7.88 (d, 4 J=2.1 Hz, 1H), 10.63 (s, 1H, OH). *13*C{*1*H} NMR (75 MHz, DMSO-d6): *δ* = 21.6 (*t*), 24.8 (*t*), 24.9 (*t*), 56.5 (*t*), 57.5 (*t*), 107.1 (*s*), 111.4 (*s*), 113.8 (*s*), 119.4 (*s*), 130.3 (*s*), 133.5 (*d*), 137.9 (*d*), 152.5 (*s*), 184.6 (*s*).

### Refinement

The C-bound H-atoms were included at calculated positions and treated using a riding model, with aromatic C—H = 0.95 Å, and methylene C—H = 0.99 Å, and *U*_iso_(H) = 1.2*U*_eq_(C). The phenolic H-atom (H0) was located in a difference Fourier and was also allowed to ride in the refinement, with *U*_iso_(H) = 1.5*U*_eq_(O).

### Crystal data

**Table:**

C_14_H_13_Br_3_NOS_2_^+^·Br^−^	*F*(000) = 1136
*M**_r_* = 595.01	*D*_x_ = 2.232 Mg m^−^^3^
Monoclinic, *P*2_1_/*c*	Melting point = 501–502 K
Hall symbol: -P 2ybc	Mo *K*α radiation, λ = 0.71073 Å
*a* = 10.484 (2) Å	Cell parameters from 17310 reflections
*b* = 7.9240 (16) Å	θ = 2.6–29.6°
*c* = 21.396 (4) Å	µ = 9.33 mm^−^^1^
β = 95.16 (3)°	*T* = 153 K
*V* = 1770.4 (6) Å^3^	Plate, colourless
*Z* = 4	0.26 × 0.13 × 0.05 mm

### Data collection

**Table:**

Stoe IPDS 2T area-detector diffractometer	4399 independent reflections
Radiation source: fine-focus sealed tube	3413 reflections with *I* > 2σ(*I*)
Graphite monochromator	*R*_int_ = 0.132
Detector resolution: 6.67 pixels mm^-1^	θ_max_ = 28.3°, θ_min_ = 2.0°
rotation method scans	*h* = −13→13
Absorption correction: for a sphere [modified Dwiggins (1975)]	*k* = −10→9
*T*_min_ = 0.047, *T*_max_ = 0.073	*l* = −28→28
19422 measured reflections	

### Refinement

**Table:**

Refinement on *F*^2^	Primary atom site location: structure-invariant direct methods
Least-squares matrix: full	Secondary atom site location: difference Fourier map
*R*[*F*^2^ > 2σ(*F*^2^)] = 0.061	Hydrogen site location: inferred from neighbouring sites
*wR*(*F*^2^) = 0.137	H-atom parameters constrained
*S* = 1.08	*w* = 1/[σ^2^(*F*_o_^2^) + (0.0656*P*)^2^] where *P* = (*F*_o_^2^ + 2*F*_c_^2^)/3
4399 reflections	(Δ/σ)_max_ < 0.001
200 parameters	Δρ_max_ = 1.52 e Å^−^^3^
0 restraints	Δρ_min_ = −1.03 e Å^−^^3^

### Special details

**Table:**

Experimental. Absorption correction: Interpolation using International Tables Vol. C, Table 6.3.3.3 for values of µR in the range 0-2.5, and International Tables Vol. II, Table 5.3.6B for µR in the range 2.6–10.0. The interpolation procedure (Dwiggins, 1975) is used with some modification.
Geometry. All e.s.d.'s (except the e.s.d. in the dihedral angle between two l.s. planes) are estimated using the full covariance matrix. The cell e.s.d.'s are taken into account individually in the estimation of e.s.d.'s in distances, angles and torsion angles; correlations between e.s.d.'s in cell parameters are only used when they are defined by crystal symmetry. An approximate (isotropic) treatment of cell e.s.d.'s is used for estimating e.s.d.'s involving l.s. planes.
Refinement. Refinement of *F*^2^ against ALL reflections. The weighted *R*-factor *wR* and goodness of fit *S* are based on *F*^2^, conventional *R*-factors *R* are based on *F*, with *F* set to zero for negative *F*^2^. The threshold expression of *F*^2^ > σ(*F*^2^) is used only for calculating *R*-factors(gt) *etc*. and is not relevant to the choice of reflections for refinement. *R*-factors based on *F*^2^ are statistically about twice as large as those based on *F*, and *R*- factors based on ALL data will be even larger.

### Fractional atomic coordinates and isotropic or equivalent isotropic displacement parameters (Å^2^)

**Table:**

	*x*	*y*	*z*	*U*_iso_*/*U*_eq_	
N	0.2558 (5)	1.1734 (8)	0.0481 (2)	0.0247 (12)	
O	0.6690 (4)	0.9340 (7)	0.1718 (2)	0.0250 (10)	
H0	0.7142	0.9925	0.1496	0.038*	
Br1	0.93336 (6)	0.89688 (11)	0.24480 (3)	0.03220 (19)	
Br2	0.68140 (7)	1.21285 (10)	0.43451 (3)	0.03022 (18)	
Br3	0.27602 (6)	0.94400 (9)	0.29348 (3)	0.02288 (16)	
Br4	0.80174 (7)	1.19802 (10)	0.09104 (3)	0.03071 (18)	
S1	0.20158 (14)	1.0731 (2)	0.16132 (7)	0.0216 (3)	
S2	0.46012 (15)	1.1582 (2)	0.13143 (7)	0.0246 (3)	
C1	0.5624 (6)	1.0722 (8)	0.2511 (3)	0.0201 (12)	
C2	0.6747 (6)	0.9999 (9)	0.2309 (3)	0.0210 (12)	
C3	0.7839 (6)	0.9945 (9)	0.2716 (3)	0.0249 (13)	
C4	0.7862 (7)	1.0585 (10)	0.3319 (3)	0.0270 (14)	
H4	0.8626	1.0542	0.3594	0.032*	
C5	0.6763 (7)	1.1286 (9)	0.3516 (3)	0.0249 (13)	
C6	0.5634 (6)	1.1376 (8)	0.3120 (3)	0.0219 (12)	
H6	0.4886	1.1871	0.3260	0.026*	
C7	0.4449 (6)	1.0855 (9)	0.2082 (3)	0.0227 (13)	
C8	0.3247 (6)	1.0477 (9)	0.2205 (3)	0.0221 (12)	
C9	0.3003 (5)	1.1413 (9)	0.1063 (3)	0.0208 (12)	
C10	0.1219 (6)	1.1466 (11)	0.0266 (3)	0.0287 (15)	
H10A	0.0709	1.1381	0.0632	0.034*	
H10B	0.1126	1.0393	0.0030	0.034*	
C11	0.0718 (7)	1.2914 (10)	−0.0152 (3)	0.0306 (15)	
H11A	0.0750	1.3977	0.0092	0.037*	
H11B	−0.0185	1.2697	−0.0309	0.037*	
C12	0.1533 (8)	1.3085 (11)	−0.0705 (3)	0.0340 (16)	
H12A	0.1453	1.2047	−0.0963	0.041*	
H12B	0.1218	1.4046	−0.0972	0.041*	
C13	0.2923 (7)	1.3368 (10)	−0.0478 (3)	0.0297 (15)	
H13A	0.3015	1.4482	−0.0270	0.036*	
H13B	0.3440	1.3383	−0.0843	0.036*	
C14	0.3436 (7)	1.2010 (11)	−0.0022 (3)	0.0291 (15)	
H14A	0.3534	1.0942	−0.0253	0.035*	
H14B	0.4291	1.2348	0.0171	0.035*	

### Atomic displacement parameters (Å^2^)

**Table:**

	*U*^11^	*U*^22^	*U*^33^	*U*^12^	*U*^13^	*U*^23^
N	0.024 (3)	0.033 (3)	0.017 (2)	−0.001 (2)	−0.0002 (19)	0.003 (2)
O	0.028 (2)	0.029 (3)	0.0191 (19)	−0.002 (2)	0.0093 (17)	−0.0033 (19)
Br1	0.0235 (3)	0.0384 (4)	0.0354 (3)	0.0043 (3)	0.0065 (3)	0.0071 (3)
Br2	0.0406 (4)	0.0313 (4)	0.0183 (3)	−0.0038 (3)	−0.0001 (2)	−0.0008 (3)
Br3	0.0253 (3)	0.0252 (3)	0.0188 (3)	−0.0002 (2)	0.0056 (2)	0.0019 (2)
Br4	0.0434 (4)	0.0259 (4)	0.0247 (3)	0.0012 (3)	0.0133 (3)	0.0004 (3)
S1	0.0211 (6)	0.0256 (8)	0.0184 (6)	−0.0011 (6)	0.0037 (5)	0.0009 (6)
S2	0.0218 (7)	0.0335 (9)	0.0186 (6)	−0.0036 (6)	0.0024 (5)	0.0035 (6)
C1	0.025 (3)	0.017 (3)	0.018 (2)	0.000 (2)	0.003 (2)	0.002 (2)
C2	0.028 (3)	0.017 (3)	0.018 (2)	−0.004 (2)	0.002 (2)	−0.001 (2)
C3	0.025 (3)	0.021 (3)	0.029 (3)	−0.002 (3)	0.008 (2)	0.002 (3)
C4	0.027 (3)	0.028 (4)	0.025 (3)	−0.002 (3)	−0.006 (2)	0.005 (3)
C5	0.036 (3)	0.023 (3)	0.015 (2)	−0.006 (3)	0.002 (2)	−0.001 (2)
C6	0.030 (3)	0.015 (3)	0.021 (3)	0.001 (2)	0.004 (2)	0.000 (2)
C7	0.031 (3)	0.020 (3)	0.017 (3)	0.001 (2)	−0.002 (2)	0.000 (2)
C8	0.028 (3)	0.022 (3)	0.016 (2)	0.003 (3)	0.001 (2)	0.000 (2)
C9	0.016 (2)	0.022 (3)	0.024 (3)	−0.001 (2)	0.002 (2)	0.000 (2)
C10	0.024 (3)	0.038 (4)	0.025 (3)	0.001 (3)	0.002 (2)	0.002 (3)
C11	0.027 (3)	0.033 (4)	0.033 (3)	0.003 (3)	0.004 (3)	0.002 (3)
C12	0.046 (4)	0.034 (4)	0.022 (3)	0.004 (3)	0.002 (3)	0.007 (3)
C13	0.043 (4)	0.028 (4)	0.018 (3)	−0.003 (3)	0.004 (3)	0.000 (3)
C14	0.030 (3)	0.040 (4)	0.017 (3)	0.004 (3)	0.004 (2)	0.004 (3)

### Geometric parameters (Å, º)

**Table:**

N—C9	1.314 (8)	C4—H4	0.9500
N—C10	1.453 (8)	C5—C6	1.394 (9)
N—C14	1.495 (8)	C6—H6	0.9500
O—C2	1.365 (7)	C7—C8	1.344 (9)
O—H0	0.8400	C10—C11	1.519 (10)
Br1—C3	1.883 (7)	C10—H10A	0.9900
Br2—C5	1.892 (6)	C10—H10B	0.9900
Br3—C8	1.876 (6)	C11—C12	1.527 (10)
Br3—Br4^i^	3.3063 (11)	C11—H11A	0.9900
S1—C9	1.723 (6)	C11—H11B	0.9900
S1—C8	1.737 (6)	C12—C13	1.511 (11)
S2—C9	1.718 (6)	C12—H12A	0.9900
S2—C7	1.761 (6)	C12—H12B	0.9900
C1—C6	1.401 (8)	C13—C14	1.518 (10)
C1—C2	1.412 (9)	C13—H13A	0.9900
C1—C7	1.472 (9)	C13—H13B	0.9900
C2—C3	1.375 (9)	C14—H14A	0.9900
C3—C4	1.385 (10)	C14—H14B	0.9900
C4—C5	1.380 (10)		
			
C9—N—C10	121.5 (5)	N—C9—S1	121.6 (5)
C9—N—C14	121.4 (5)	S2—C9—S1	116.1 (4)
C10—N—C14	115.7 (5)	N—C10—C11	110.5 (6)
C2—O—H0	109.5	N—C10—H10A	109.6
C8—Br3—Br4^i^	169.9 (2)	C11—C10—H10A	109.6
C9—S1—C8	94.7 (3)	N—C10—H10B	109.6
C9—S2—C7	95.7 (3)	C11—C10—H10B	109.6
C6—C1—C2	119.9 (6)	H10A—C10—H10B	108.1
C6—C1—C7	119.3 (6)	C10—C11—C12	109.6 (6)
C2—C1—C7	120.8 (5)	C10—C11—H11A	109.7
O—C2—C3	122.7 (6)	C12—C11—H11A	109.7
O—C2—C1	118.1 (5)	C10—C11—H11B	109.7
C3—C2—C1	119.2 (6)	C12—C11—H11B	109.7
C2—C3—C4	121.5 (6)	H11A—C11—H11B	108.2
C2—C3—Br1	119.2 (5)	C13—C12—C11	110.8 (6)
C4—C3—Br1	119.2 (5)	C13—C12—H12A	109.5
C5—C4—C3	119.1 (6)	C11—C12—H12A	109.5
C5—C4—H4	120.4	C13—C12—H12B	109.5
C3—C4—H4	120.4	C11—C12—H12B	109.5
C4—C5—C6	121.5 (6)	H12A—C12—H12B	108.1
C4—C5—Br2	118.3 (5)	C12—C13—C14	112.2 (6)
C6—C5—Br2	120.2 (5)	C12—C13—H13A	109.2
C5—C6—C1	118.8 (6)	C14—C13—H13A	109.2
C5—C6—H6	120.6	C12—C13—H13B	109.2
C1—C6—H6	120.6	C14—C13—H13B	109.2
C8—C7—C1	127.4 (6)	H13A—C13—H13B	107.9
C8—C7—S2	114.9 (5)	N—C14—C13	111.2 (6)
C1—C7—S2	117.7 (5)	N—C14—H14A	109.4
C7—C8—S1	118.7 (5)	C13—C14—H14A	109.4
C7—C8—Br3	126.2 (5)	N—C14—H14B	109.4
S1—C8—Br3	114.7 (4)	C13—C14—H14B	109.4
N—C9—S2	122.3 (5)	H14A—C14—H14B	108.0
			
C6—C1—C2—O	178.5 (6)	S2—C7—C8—S1	0.2 (8)
C7—C1—C2—O	−3.8 (9)	C1—C7—C8—Br3	−7.4 (11)
C6—C1—C2—C3	0.1 (10)	S2—C7—C8—Br3	172.4 (4)
C7—C1—C2—C3	177.9 (6)	C9—S1—C8—C7	0.8 (6)
O—C2—C3—C4	−178.4 (6)	C9—S1—C8—Br3	−172.3 (4)
C1—C2—C3—C4	−0.1 (10)	Br4^i^—Br3—C8—C7	−87.8 (13)
O—C2—C3—Br1	1.3 (9)	Br4^i^—Br3—C8—S1	84.7 (12)
C1—C2—C3—Br1	179.6 (5)	C10—N—C9—S2	175.4 (6)
C2—C3—C4—C5	0.3 (11)	C14—N—C9—S2	9.5 (10)
Br1—C3—C4—C5	−179.4 (5)	C10—N—C9—S1	−2.5 (10)
C3—C4—C5—C6	−0.5 (11)	C14—N—C9—S1	−168.4 (6)
C3—C4—C5—Br2	179.3 (5)	C7—S2—C9—N	−176.5 (6)
C4—C5—C6—C1	0.6 (11)	C7—S2—C9—S1	1.5 (5)
Br2—C5—C6—C1	−179.2 (5)	C8—S1—C9—N	176.6 (6)
C2—C1—C6—C5	−0.4 (10)	C8—S1—C9—S2	−1.4 (5)
C7—C1—C6—C5	−178.1 (6)	C9—N—C10—C11	138.2 (7)
C6—C1—C7—C8	−47.4 (10)	C14—N—C10—C11	−55.1 (8)
C2—C1—C7—C8	134.8 (8)	N—C10—C11—C12	57.3 (8)
C6—C1—C7—S2	132.8 (6)	C10—C11—C12—C13	−57.8 (9)
C2—C1—C7—S2	−44.9 (8)	C11—C12—C13—C14	54.4 (9)
C9—S2—C7—C8	−1.0 (6)	C9—N—C14—C13	−142.7 (7)
C9—S2—C7—C1	178.8 (5)	C10—N—C14—C13	50.6 (9)
C1—C7—C8—S1	−179.6 (6)	C12—C13—C14—N	−49.1 (8)

Symmetry code: (i) −*x*+1, *y*−1/2, −*z*+1/2.

### Hydrogen-bond geometry (Å, º)

**Table:**

*D*—H···*A*	*D*—H	H···*A*	*D*···*A*	*D*—H···*A*
O—H0···Br4	0.84	2.30	3.120 (5)	167
C6—H6···O^ii^	0.95	2.56	3.424 (8)	151
C10—H10*A*···S1	0.99	2.46	2.985 (7)	113
C13—H13*A*···Br2^ii^	0.99	2.88	3.836 (7)	163
C14—H14*B*···S2	0.99	2.51	3.026 (7)	112

Symmetry code: (ii) −*x*+1, *y*+1/2, −*z*+1/2.

## References

[bb1] Birsa, M. L. & Ganju, D. (2003). *J. Phys. Org. Chem.* **16**, 207–212.

[bb2] Bryce, M. R. (2000). *J. Mater. Chem.* **10**, 589–598.

[bb3] Dwiggins, C. W. (1975). *Acta Cryst.* A**31**, 146–148.

[bb4] Frasch, M., Mono, S., Pritzkow, H. & Sundermeyer, W. (1993). *Chem. Ber.* **126**, 273–275.

[bb5] Narita, M. & Pittman, C. U. Jr (1976). *Synthesis*, pp. 489–514.

[bb6] Sheldrick, G. M. (2008). *Acta Cryst.* A**64**, 112–122. 10.1107/S0108767307043930 18156677

[bb7] Stoe & Cie (2002). *X-AREA* Stoe & Cie, Darmstadt, Germany.

